# Supplementary material for: Validation of a Food Frequency Questionnaire to Assess Intake of n-3 Polyunsaturated Fatty Acids in Switzerland
Source: Nutrients. 2019 Aug 10;11(8):1863. doi: 10.3390/nu11081863 (PMC6722517; doi:10.3390/nu11081863)
Supplement: Supplementary file 1 [file nutrients-11-01863-s001.pdf]

OMEGA-3 FATTY ACIDS QUESTIONNAIRE

ID: ___________________________

Date of survey: ___________________________

1) Sex

☐ Male ☐ Female

2) Year of birth: ________________________

3) How many times in the past six months have you consumed fish or seafood? (including sushi prepared with fish or seafood)

☐ Never	☐ Twice a week
☐ Less than one a month	☐ 3-4 times per week
☐ Once a month	☐ 5-6 times per week
☐ 2-3 times per month	☐ Once a day
☐ Once a week	☐ Multiple times per day

→ If your answer was NEVER, go to question 7.

4) If you ate fish or seafood, how much of it did you eat?

☐ Less than a filet or less than four pieces of sushi

☐ About one filet or 4-14 pieces of sushi

☐ More than one filet or more than 14 pieces of sushi

5) In what way did you eat fish or seafood?

☐ Fish fingers/deep fried

☐ Sushi

☐ Fresh fish

☐ Frozen fish

☐ Fish-burger

6) Please tick all the fish and seafood you consume the most.

- ☐ Salmon
- ☐ Pangasius
- ☐ European perch
- ☐ Trout
- ☐ Tuna
- ☐ Gilt-head bream
- ☐ Halibut
- ☐ Herring
- ☐ European plaice
- ☐ Mackerel
- ☐ Lemon sole
- ☐ Pike
- ☐ Sardines
- ☐ (Common) sole
- ☐ Carp
- ☐ Cod
- ☐ Flounder
- ☐ Perch
- ☐ Turbot
- ☐ Coregonus (blue whitefish)
- ☐ Salvelinus/char
- ☐ Anchovies
- ☐ Monkfish
- ☐ Surimi
- ☐ Zander
- ☐ Caviar
- ☐ Lobster
- ☐ Octopus/Calamari
- ☐ Scallops
- ☐ Oysters
- ☐ Crab
- ☐ Shrimps/Prawns/Scampi
- ☐ ________________________________

7) How many times in the past six months did you eat walnuts?

- ☐ Never
- ☐ Twice a week
- ☐ Less than one a month
- ☐ 3-4 times per week
- ☐ Once a month
- ☐ 5-6 times per week
- ☐ 2-3 times per month
- ☐ Once a day
- ☐ Once a week
- ☐ Multiple times per day

→ If your answer was NEVER, go to question 9.

8) If you ate walnuts, how many did you eat?

- ☐ Less than 10 nut halves (< 30 grams)
- ☐ 10-20 nut halves (30-60 grams)
- ☐ More than 20 nut halves (> 60 grams)

9) How many times in the past six months have you used rapeseed oil?

- ☐ Never
- ☐ Twice a week
- ☐ Less than one a month
- ☐ 3-4 times per week
- ☐ Once a month
- ☐ 5-6 times per week
- ☐ 2-3 times per month
- ☐ Once a day
- ☐ Once a week
- ☐ Multiple times per day

→ If your answer was NEVER, go to question 11.

10) If you used rapeseed oil, how much of it did you use?

- ☐ Less than a teaspoon
- ☐ 1-2 teaspoons
- ☐ 1 tablespoon
- ☐ 1-2 tablespoons
- ☐ More than two tablespoons

11) How many times in the past six months did you eat flaxseed?

☐ Never	☐ Twice a week
☐ Less than one a month	☐ 3-4 times per week
☐ Once a month	☐ 5-6 times per week
☐ 2-3 times per month	☐ Once a day
☐ Once a week	☐ Multiple times per day

→ If your answer was NEVER, go to question 13.

12) If you ate flaxseed, how much of it did you eat?

- ☐ Less than a teaspoon
- ☐ 1-2 teaspoons
- ☐ 1 tablespoon
- ☐ 1-2 tablespoons
- ☐ More than two tablespoons

13) How many times in the past six months did you use linseed oil?

☐Never	☐ Twice a week
☐ Less than one a month	☐ 3-4 times per week
☐ Once a month	☐ 5-6 times per week
☐ 2-3 times per month	☐ Once a day
☐ Once a week	☐ Multiple times per day

→ If your answer was NEVER, go to question 15.

14) If you used linseed oil, how much of it did you use?

- ☐ Less than a teaspoon
- ☐ 1-2 teaspoons
- ☐ 1 tablespoon
- ☐ 1-2 tablespoons
- ☐ More than two tablespoons

15) How many times in the past six months did you eat chia seeds?

☐Never ☐ Twice a week

☐ Less than one a month ☐ 3-4 times per week

☐ Once a month ☐ 5-6 times per week

☐ 2-3 times per month ☐ Once a day

☐ Once a week ☐ Multiple times per day

→ If your answer was NEVER, go to question 17.

16) If you ate chia seeds, how much of them did you eat?

☐ Less than a teaspoon

☐ 1-2 teaspoons

☐ 1 tablespoon

☐ 1-2 tablespoons

☐ More than two tablespoons

17) How many times in the past six months did you use vegetable margarine?

☐Never ☐ Twice a week

☐ Less than one a month ☐ 3-4 times per week

☐ Once a month ☐ 5-6 times per week

☐ 2-3 times per month ☐ Once a day

☐ Once a week ☐ Multiple times per day

→ If your answer was NEVER, go to question 20.

18) If you used vegetable margarine, how much of it did you use?

☐ Less than a teaspoon

☐ 1-2 teaspoons

☐ 1 tablespoon

☐ 1-2 tablespoons

☐ More than two tablespoons

19) Which margarine (brand and product) did you use?

____

20) Have you taken an omega-3 fatty acid supplement or a fish oil supplement at least once a week in the last six months?

- ☐ No
- ☐ Yes

→ If your answer was NO, go to question 23.

21) How many times did you take the supplement or fish oil?

- ☐ Once per week
- ☐ 2-3 times per week
- ☐ 4-5 times per week
- ☐ daily
- ☐ Multiple times per day

22) Please write down the name and brand (dose if known) of the products:

____

23) Have you consumed an omega-3 enriched products in the past six months that was not mentioned in the questionnaire?

- ☐ No
- ☐ Yes

→ If your answer was NO, the questionnaire ends here for you.

24) Which product did you consume and how often did you consume it?

____

If you have any comments about the questionnaire in general or about your diet in the last six months, you can write them down here:

____

____

____

____

____

____

____

____
